# Local monitoring of SARS-CoV-2 variants in two large California counties in 2021

**DOI:** 10.1038/s41598-022-21481-0

**Published:** 2022-10-11

**Authors:** Noah Kojima, Eugenia Khorosheva, Lauren Lopez, Mikhail Hanewich-Hollatz, J. Cesar Ignacio-Espinoza, Matthew Brobeck, Janet Chen, Matthew Geluz, Victoria Hess, Sophia Quasem, Nabjot Sandhu, Elias Salfati, Maria Shacreaw, George Way, Zhiyi Xie, Vladimir Slepnev, Jeffrey D. Klausner

**Affiliations:** 1 Department of Medicine, University of California Los Angeles, 10833 Le Conte Ave, Los Angeles, CA 90095 USA; 2 Curative Inc., San Dimas, CA USA; 3 Department of Population and Public Health Sciences, Keck School of Medicine, University of Southern California, Los Angeles, 90033 USA

**Keywords:** Microbiology, Diseases

## Abstract

Coronavirus Disease 2019 (COVID-19), caused by the severe acute respiratory syndrome coronavirus 2 (SARS-CoV-2), continues to persist due to mutations resulting in newer, more infectious variants of concern. We aimed to leverage an ongoing private SARS-CoV-2 testing laboratory’s infrastructure to monitor SARS-CoV-2 variants in two large California counties. Study enrollment was offered to adults aged 18 years or older in Los Angeles County and Riverside County who recently tested positive for SARS-CoV-2 with a polymerase chain reaction (PCR) assay. A cycle threshold value less than or equal to 30 cycles was considered a positive test for sequencing purposes. Within 5 days of study enrollment, clinician-monitored, self-collected oral fluid and anterior nares swab specimens were obtained from participants. Specimens were transported and stored at 8 °C or cooler. Samples underwent ribonucleic acid extraction, library preparation, and sequencing. SARS-CoV-2 lineages were identified using sequencing data. Participant and genomic data were analyzed using statistical tools and visualized with toolkits. The study was approved by Advarra Institutional Review Board (Pro00053729). From May 27, 2021 to September 9, 2021, 503 individuals were enrolled and underwent specimen collection. Of the 503 participants, 238 (47.3%) participants were women, 329 (63.6%) participants were vaccinated, and 221 (43.9%) participants were of Hispanic or Spanish origin. Of the cohort, 496 (98.6%) participants had symptoms at the time of collection. Among the 503 samples, 443 (88.1%) nasal specimens and 353 (70.2%) oral specimens yielded positive sequencing results. Over our study period, the prevalence of the Alpha variant of SARS-CoV-2 decreased (initially 23.1% [95% confidence interval (95% CI): 0–0.49%] to 0% [95% CI 0.0–0.0%]) as the prevalence of the Delta variant of SARS-CoV-2 increased (initially 33.3% [95% CI 0.0–100.0%] to 100.0% [95% CI 100.0–100.0%]). A strain that carried mutations of both Delta and Mu was identified. We found that outpatient SARS-CoV-2 variant surveillance could be conducted in a timely and accurate manner. The prevalence of different variants changed over time. A higher proportion of nasal specimens yielded results versus oral specimens. Timely and regional outpatient SARS-CoV-2 variant surveillance could be used for public health efforts to identify changes in SARS-CoV-2 strain epidemiology.

## Introduction

Coronavirus disease 2019 (COVID-19) cases, caused by the severe acute respiratory syndrome coronavirus 2 (SARS-CoV-2), continue to persist. Like many viruses, SARS-CoV-2 mutates^1^. When those mutations occur in key parts of important proteins, there is the potential for the virus to become more infectious, resistant to treatments, and able to avoid immunity that could be due to vaccination or natural infection^2,3^.

Following widespread infections caused by the wildtype strain of SARS-CoV-2, the first identified variant of concern was the Alpha variant (UK variant), which spread approximately 50% faster than the wildtype strain^2^. Following the Alpha variant, the Beta (South Africa) and Gamma (Brazilian) variants of concern were identified^4^. The Delta variant of SARS-CoV-2 followed becoming the dominant variant of concern in the United States^5^, with new variants actively being identified^6^. Compared to the wildtype strain of SARS-CoV-2, the Delta variant is more infectious and has the potential to escape vaccination-medicated immune protection^2,7–9^.

Due to the major public health concern of SARS-CoV-2, surveillance programs are needed to monitor SARS-CoV-2 variants to inform public health efforts. With the development of new sequencing tools, it is possible for genomic data to be used to inform those public health responses as novel mutations are identified in widely circulating strains of SARS-CoV-2. The World Health Organization proposed that a strong and resilient global sequencing network that provides useable and timely results is needed to maximize the public health impact of sequencing^10^. Regarding that important effort, we aimed to leverage an ongoing private SARS-CoV-2 testing laboratory’s infrastructure to monitor SARS-CoV-2 variants in two large counties in California.

## Methods

### Enrollment

Study enrollment was offered to eligible adults (18 years or older) in Los Angeles County and Riverside County who tested positive for SARS-CoV-2 by a reverse transcription polymerase chain reaction (RT-PCR) assay conducted at a private laboratory (Curative, San Dimas, CA). To meet eligibility criteria, potential participants were required to have a positive SARS-CoV-2 RT-PCR result with a cycle threshold value less than or equal to 30 cycles within 5 days of study enrollment and time of sample collection for sequencing. Vulnerable subjects, i.e., pregnant women, nursing home residents or other institutionalized people, prisoners, and persons without decisional capacity, were excluded from the study. Written informed consent was obtained from each participant prior to study enrollment. Demographic information and vaccination status was collected from participants with a survey.

### Specimen collection and transport

Trained healthcare workers directly observed participants as they self-collecting oral fluid and anterior nares swab specimens. Specimens placed into 10 mL collection tubes containing DNA/RNA Shield Stabilization Solution (Zymo Research, Irvine, CA). Samples were transported to the laboratory at 2–8 °C within 5 h of specimen collection and stored at 4 °C for up to 7 days before library preparation.

### Ribonucleic acid (RNA) isolation

RNA extraction was performed using silica-based. Either manual silica column-based extraction (Total RNA Purification Kit 96 Deep Well Plate Format Dx, Norgen Biotek Corp., Thorold, ON) or a modified automated magnetic silica beads-based extraction method (Thermo Scientific KingFisher™ Flex, Thermo Scientific, Waltham, MA) were used for extraction. RNA was eluted in 60 μL of 10 mM Tris (pH 7.4).

### Library preparation and sequencing

The libraries were prepared using an Illumina COVIDSeq protocol on 96-well plates (Illumina, Inc., San Diego, CA). Samples were processed alongside a positive control (SARS-CoV-2 BEI NR-52287 genotype A) and a negative control of human nasal specimen without SARS-CoV-2 RNA. An indexed sample library from each plate was pooled and quantified using a fluorometer (Qubit 3.0, Invitrogen, Waltham, MA). Four 96-well plates were combined at equimolar concentrations and sequenced. Dilution and loading were performed per the manufacturer instruction (Illumina, Inc., San Diego, CA). Dual-indexed paired-end sequencing was performed to get a deeper sequencing depth. Sequencing aimed to have 1–2 million reads per sample (NextSeq2000, Illumina, Inc., San Diego, CA).

### Quality control of reads

Paired-end reads were filtered and trimmed to eliminate the presence of primers and adapters. Data quality was assessed to ensure high-quality reads. A minimum quality score of 30 was selected (*pTrimer-1.3.4 -a Primers.bed -q 30 -t pair*)^11^.

### Mapping

Paired-end reads were then mapped against the ‘Wuhan seafood market pneumonia virus isolate” Wuhan-Hu-1 genome (*Accession number: NC_045512.2*)^12^, using the *bwa* software package^13^. Each read was aligned using the software package, then alignments were paired. Alignment files were then subsetted to consider only proper pairs with a quality score greater than 12^14^.

### Variant identification

Variable sites were generated, regardless of coverage depth assuming a haploid genome using the bcftools software package^14^. Variable sites were then filtered for quality and coverage. Finally, the consensus genome was generated (CoSa suite, Pacific Biosciences, Menlo Park, CA). Consensus genomes assessed with pangolin and nextclade^15^.

### Ad-hoc analysis

Custom scripts were used to calculate sequencing, effort, and the percentage of reads used to assemble de novo genomes and base pair coverage. These can be found at (https://github.com/curative/bioinformatics).

### SARS-CoV-2 prevalence in Los Angeles and Riverside counties

Pooled de-identified SARS-CoV-2 test from Los Angeles County and Riverside County was plotted over time to assess SARS-CoV-2 prevalence during our study period and compared to country data that included test results from all testing sites that partnered with the county. Results are displayed in a figure of percentage positivity, i.e., the number of positive tests over a total number of tests conducted.

### Analysis of consensus sequences mutations and identifying similar isolates

A lineage comparison was done using the Outbreak.info resource (Scripps Research, La Jolla, CA 92037). A search for genomic sequences similar to the identified SARS-CoV-2 isolates was performed using Nucleotide BLAST 2.6.0+. Isolated were compared to all sequences available on GISAID (https://www.epicov.org/epi3/cfrontend#2c08bd). Data underwent alignment to identify gaps in generated consensus sequences and to match them with positions in amino acid sequences carrying hallmark mutations using Geneious Prime software (Geneious, Auckland, New Zealand).

### Data availability and materials

Results were submitted to the Global Influenza Surveillance and Response System (GISAID) EpiCoV database for widespread data sharing and surveillance, a public surveillance service^16,17^. All results were submitted to the GISAID EpiCoV database.

The accession numbers that were added are listed in Supplemental Table 5 and are publicly available. The datasets generated and/or analyzed during the current study are available in the GISAID EpiCoV database repository, https://gisaid.org/.

### Human subjects

The study was approved by Advarra Institutional Review Board under Pro00053729 on May 10, 2021. All research was performed in accordance with relevant regulations in accordance with the Declaration of Helsinki. Informed consent was obtained from all participants. No vulnerable groups were included in this study.

## Results

**Figure 1:**
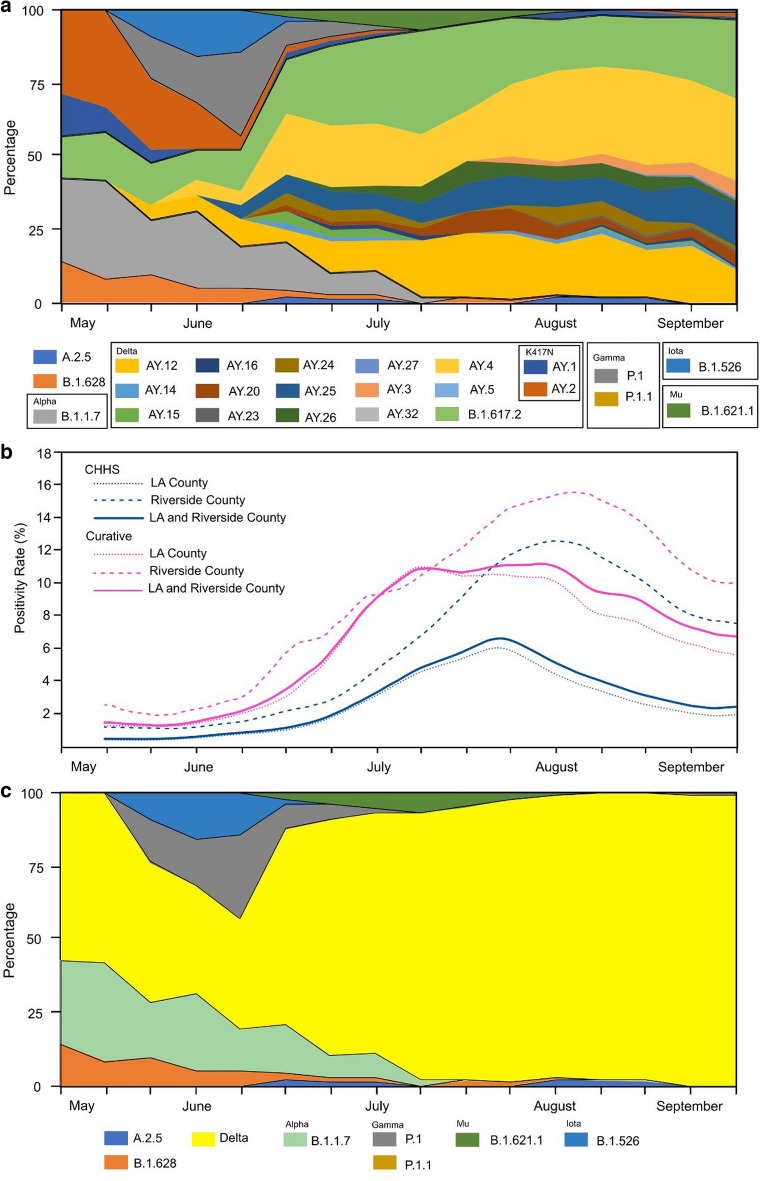
(**a**) SARS-CoV-2 expanded lineage data over time from a cohort of outpatient participants in Los Angeles County and Riverside County, California, May 27, 2021 to September 9, 2021 (n = 503). (**b**) Total SARS-CoV-2 positivity rates detected in a private laboratory in Los Angeles County and Riverside County, California, May 27, 2021 to September 9, 2021 (n = 503). (**c**) SARS-CoV-2 lineage data by variant over time from a cohort of outpatient participants in Los Angeles County and Riverside County, California, May 27, 2021 to September 9, 2021 (n = 503). *Lineages with the K417N substitution were replaced by different Delta lineages. Data was smoothed over three consecutive weeks.

From May 27, 2021 to September 9, 2021, 503 SARS-CoV-2 PCR positive participants were enrolled and underwent specimen collection. Participants were enrolled over several months of the study period (Supplemental Table 1). Over the study period, laboratory percentage positivity data was compared to county positivity, the positivity of SARS-CoV-2 tests were similar temporally (Fig. 1b). Of the 503 participants, 238 (47.3%) participants were women, 329 (63.6%) participants were vaccinated, and 221 (43.9%) participants were of Hispanic or Spanish origin (Table 1). Of the cohort, 496 (98.6%) participants had symptoms at time of collection with congestion, cough, and fatigue being most common. Among the 503 samples, 443 (88.1%) nasal specimens and 353 (70.2%) oral specimens yielded positive sequencing results.

**Table 1:** Demographic characteristics, clinical symptoms and SARS-CoV-2 lineage data from a cohort of outpatient participants in Los Angeles County and Riverside County, California, May 27, 2021 to September 9, 2021 (n = 503).

Variable	Mean/number	Standard deviation or percent
Age, years	40.9	12.6
Gender, female	238	47.3%
Days to collection	2.8	0.7
**Ethnicity**		
Not disclosed	25	5.0%
Black or African American	1	0.2%
Hispanic or Spanish origin	221	43.9%
Not Hispanic or Spanish origin	208	41.4%
Prefer not to share	48	9.5%
**Vaccinated**		
No	182	36.2%
Yes	320	63.6%
Declined to disclose	1	0.2%
**Vaccine manufacturer**		
J&J	56	16.6%
Moderna	98	29.1%
Pfizer	179	53.1%
Pfizer and AstraZeneca	1	0.3%
Sinopharm	3	0.9%
**Symptoms**		
Congestion or runny nose	375	75.6%
Cough	368	74.2%
Fatigue	343	69.2%
Headache	301	60.7%
Muscle or body aches	290	58.5%
New loss of taste or smell	289	58.3%
Fever or chills	220	44.4%
Sore throat	216	43.5%
Shortness of breath	125	25.2%
Diarrhea	105	21.2%
Nausea or vomiting	80	16.1%
Bleeding	1	0.2%
Eye redness or dry mouth	1	0.2%
Stomach pain	1	0.2%

Over our study period, the prevalence of the Alpha variant of SARS-CoV-2 decreased (initially 23.1% [95% confidence interval (95% CI) 0–0.49%] to 0% [95% CI 0.0–0.0%]) as the prevalence of the Delta variant of SARS-CoV-2 increased (initially 33.3% [95% CI 0.0–100.0%] to 100.0% [95% CI 100.0–100.0%]; Fig. 1a,c, and Supplemental Table 2). SARS-CoV-2 lineages AY.4 and AY.12 made up most of the identified Delta variant. Changes of SARS-CoV-2 lineage were similar among those vaccinated and unvaccinated (Supplemental Fig. 1a,b). Cycle threshold values were similar between those vaccinated and unvaccinated at time of collection (Supplemental Table 4).

As of 29 October 2021, there were 2,110,018 reported sequences for all SARS-CoV-2 Delta lineages, with 829,064 reported sequences belonging to the B.1.617.2 lineage. Over 63% of samples originally reported as B.1.617.2 had at least one gap in their consensus sequence. The most common mutation was in the G29422T position.

During our period of surveillance, we also found a unique case of a non-identifiable isolate (hCoV-19/USA/CA-Curative-707962712299/2021) collected on July 7, 2021 sequenced on July 14, 2021 (Supplemental Table 2, Supplemental Figs. 2, 3a,b, and 4). The non-identifiable isolate had 95.79% non-N coverage. The non-identifiable isolate carried hallmark S protein mutations from Alpha, Beta, Delta, and Mu lineages of SARS-CoV-2 simultaneously, as well as rare mutations detected in AY.20 sub-lineage of Delta (Supplemental Figs. 2, 3a,b, and 4). Initially the non-identifiable isolate was classified as the B.1.621.1 lineage (Mu) using PANGO version 1.2.36. However, there is no known lineage that matches the non-identifiable isolate with PANGO version 3.1.11. BLAST alignment of the isolate consensus against the GISAID SARS-CoV-2 sequences revealed that this isolate is highly similar to > 20 cases detected in Mexico starting June 13, 2021 and 7 isolates sequenced in the United Stated either by this private laboratory or the United States Centers for Disease Control and Prevention (Supplemental Fig. 3a,b).

## Discussion

We found that outpatient SARS-CoV-2 variant surveillance could be conducted in a private laboratory in a timely and accurate manner. SARS-CoV-2 positivity rates from specimens tested in a private laboratory were similar to county level data collected during the same periods. Among a sample of people that tested positive for SARS-CoV-2, we observed that the Alpha variant of SARS-CoV-2 was most prevalent in Los Angeles and Riverside County in May 2021, however the Delta variant of SARS-CoV-2, mainly the AY.4 and AY.12 strains, became dominant over a period of weeks. Among the isolates identified as a SARS-CoV-2 Delta variant, a large number of isolates carried mutations classifying them as a sub-lineages of Delta, which is evidence of continued mutation. When compared to publicly available data on SARS-CoV-2 variants, our surveillance sampling found similar proportions of variants of concern in our outpatient samples over time^18^.

The identification of new SARS-CoV-2 variants in a timely manner is critical to public health. While it is hard to prognosticate the future, it is possible to establish a method to prioritize research of new identified virus lineages that carry novel mutations on genetic coding segments of key proteins, like the SARS-CoV-2 spike protein^19,20^. Faster identification of new SARS-CoV-2 variants of concern and understanding the rates in their change of prevalence could be critical predictors of new waves of SARS-CoV-2 and met with changes in public health recommendations. This study demonstrates that private laboratories can have a role in the surveillance of SARS-CoV-2 variants of concern.

The sheer number of people who have been infected and the total SARS-CoV-2 infected person-time has led to the rapid evolution of SARS-CoV-2. Local epidemics of populous areas creates a situation in which many new mutations can form due to the large amount of viral replication over a short period of time. It is essential that all global SARS-CoV-2 epidemics are controlled to limit the rate of new SARS-CoV-2 mutations.

Temporally, it can be observed that the Delta variant of SARS-CoV-2 arose following *en masse* vaccination efforts. There are many reported cases of breakthrough SARS-CoV-2 infections among people who are fully vaccinated. Many of those people had high viral loads (cycle threshold values less than 30), and with cycle threshold values similar when comparing those who were vaccinated to those who were not vaccinated^21,22^. Reassuringly, most breakthrough infections were mild or asymptomatic^23^. Additionally, only small differences were observed in vaccine effectiveness against symptomatic disease or death when comparing the Delta to Alpha variant with the BNT162b2 vaccine (93.7% with Alpha and 88.0% for Delta) and ChAdOx1 nCoV-19 vaccine (74.5% with Alpha and 67.0% for Delta)^24^. Due to the erosion of vaccine efficacy against the SARS-CoV-2 Delta variant, it is possible that vaccinations may have played a role in the selective pressure for the Delta variant prevalence in areas with high vaccination rates^7,9,20,25^.

In our efforts to monitor SARS-CoV-2 variants of concern, we sequenced a non-identifiable isolate of SARS-CoV-2, the hCoV-19/USA/CA-Curative-707962712299/2021 isolate. The non-identifiable isolate simultaneously carried classic S protein mutations present in the Delta variant, while also displaying hallmark S protein mutations observed in Alpha, Beta, Mu and Kappa variants of SARS-CoV-2. The non-identifiable isolate carried seven S protein mutations prevalent in Mu variant, notably the S:R346K amino acid substitution in the Receptor Binding Domain and two mutations of concern/interest, S:E484K and S:N501Y, in the Receptor-Binding Motif^26^, which are also normally absent in Delta and Delta plus variants. Also, the non-identifiable isolate carried 7 mutations prevalent in AY.20 sub-lineage of Delta, including P681R mutation special for Delta lineage and known to facilitate the spike protein cleavage, enhance cell-level infectivity and pathogenicity^19^. It carried other common Delta mutations (S:T19R, S:T478K) that are not prevalent in Mu variants. Notably, the isolate carries mutations affecting epitopes for all three main classes of neutralizing antibodies (Class 1-N501Y, T478K; Class 2-E484K, L452; and Class 3-R346K), which brings concerns that this isolate might have evolutionary advantages similar to the Delta variant of SARS-CoV-2 with its ability to evade the immune system of vaccinated persons and have increased infectivity^7,27^.

The study had the following limitations. Given that our study population consisted of people undergoing outpatient SARS-CoV-2 testing, it is possible that the variants we identified are less likely to cause critical illness due to our sampling methodology. The study was conducted in Los Angeles and Riverside Counties over a short period of time, therefore there are temporal and geographic constraints of this study. Reclassification of SARS-CoV-2 variants may change our results. We believe it is likely that the isolates we sequenced will be further reclassified as new lineages or have features of interest/concern as governing bodies determine new SARS-CoV-2 variants of concern in the future.

## Conclusion

We found that outpatient SARS-CoV-2 variant surveillance could be conducted in a private laboratory in a timely and accurate manner. A higher proportion of nasal specimens yielded results than oral specimens. We observed that among a sample of people that tested positive for SARS-CoV-2, initially the Alpha variant was most prevalent in May, however the Delta variant of SARS-CoV-2 quickly became the most prevalent over a period of weeks. When compared to publicly available data on SARS-CoV-2 variants, our surveillance sampling found similar proportions of variants of concern in our outpatient samples over time. We identified a potentially new non-identifiable isolate was identified in our study cohort through surveillance measures. This study demonstrates that timely outpatient SARS-CoV-2 variant surveillance could be used for public health efforts to identify changes in SARS-CoV-2 strains in local epidemics. Government agencies should engage private laboratories in the surveillance of diseases that threaten the public’s health to supplement national disease reporting networks.

## Supplementary Information

Supplementary Information.
