# Supplementary material for: Local monitoring of SARS-CoV-2 variants in two large California counties in 2021
Source: Sci Rep. 2022 Oct 11;12:17046. doi: 10.1038/s41598-022-21481-0 (PMC9553084; doi:10.1038/s41598-022-21481-0)
Supplement: Supplementary file 1 — Supplementary Information. [file 41598_2022_21481_MOESM1_ESM.doc]

**Supplemental material**

**Text**

*Further description of a non-identifiable isolate (hCoV-19/USA/CA-Curative-707962712299/2021).*

The isolate had two uncommon in Delta amino acid substitutions were also detected together in Spike Protein V1104L and V1177I. When compared to all other known strains, the lineages where Spike Protein V1104L and V1177I were detected with a frequency of roughly 0.001% over the last 60-600 days (Supplementary Figure 3ab). The non-identifiable isolate also carried 7 matched S protein mutations that have been observed in the Mu variant, including specific hallmark mutations R346K, E484K, N501Y in the Receptor Binding Domain. The non-identifiable isolate also carried 7 mutations are prevalent in AY.20 lineage, including P681R mutation special for Delta lineage and linked to as well as other common Delta mutations T19R, T478K, D614G, D950N that are not prevalent in Mu variant.

**Supplemental Table 1.** Number of participants enrolled each month in Los Angeles County and Riverside County, Southern California, May 27, 2021 to September 9, 2021

| **Time Period** | **Participant Count** | **Percent** |
| --- | --- | --- |
| May 27, 2021 - May 31, 2021 | 7 | 1.4% |
| June 1, 2021 - June 30, 2021 | 39 | 7.8% |
| July 1, 2021 - July 31, 2021 | 106 | 21.1% |
| August 1, 2021 - August 31 | 264 | 52.5% |
| September 1, 2021 - September 17, 2021 | 87 | 17.3% |

**Supplemental Table 2.** All SARS-CoV-2 lineages identified in nasal specimens in Los Angeles County and Riverside County, Southern California, May 27, 2021 to September 9, 2021 (n= 545)

| **Lineage** | **Number** | **Percent** |
| --- | --- | --- |
| AY.4 | 110 | 21.9 |
| B.1.617.2 | 101 | 20.1 |
| AY.12 | 68 | 13.5 |
| None | 52 | 10.3 |
| AY.25 | 41 | 8.2 |
| AY.20 | 14 | 2.8 |
| AY.26 | 14 | 2.8 |
| AY.24 | 13 | 2.6 |
| AY.3 | 13 | 2.6 |
| B.1.1.7 | 13 | 2.6 |
| N/A | 11 | 2.2 |
| AY.2 | 8 | 1.6 |
| P.1 | 8 | 1.6 |
| AY.1 | 6 | 1.2 |
| AY.14 | 5 | 1.0 |
| B.1.621.1 | 5 | 1.0 |
| A.2.5 | 4 | 0.8 |
| B.1.628 | 4 | 0.8 |
| AY.15 | 3 | 0.6 |
| AY.16 | 3 | 0.6 |
| B.1.526 | 3 | 0.6 |
| AY.23 | 2 | 0.4 |
| AY.27 | 1 | 0.2 |
| P.1.10 | 1 | 0.2 |

**Supplemental Table 3.** Spike Protein AA substitutions in hCoV-19/USA/CA-Curative-707962712299/2021 - comparison with two variants with the most similar mutation sets

| ***Spike Protein AA substitutions in hCoV-19/USA/CA-Curative-707962712299/2021 - comparison with two variants with the most similar mutation sets*** | | |
| --- | --- | --- |
|  | ***Prevalent*** | ***Rare in the matching variant*** |
| ***Shared with AY.20 variant - 7 common mutations and two additional mutations*** | ***T19R, L452R, T478K, D614G, P681R, D950N, V1104L,*** *and* *T95I (69.9% of all sequences),* | *R346K (<0.001% of all isolates), E484K (<0.001% of all isolates, N501Y (<0.001% of all isolates), and V1177I (9.5% of all isolates)* |
| ***Shared with Mu variant - 7 common mutations*** | ***T95I, R346K, T478K*** ***E484K, N501Y, D614G, D950N,*** | *T19R ( <0.005% of all isolates),  L452R (~0.001% of all isolates)****,*** *T478K (<0.001% of all isolates),* *L452R( <0.005% of all isolates)****,*** *P681R (~0.001% of all isolates)****,*** *V1104L (<0.001% of all isolates)* |

**Supplemental Table 4.** Cycle threshold values for samples from unvaccinated and fully vaccinated participants enrolled in Los Angeles County and Riverside County, Southern California, May 27, 2021 to September 9, 2021

| **Cycle threshold values** | **Vaccinated (n=320)** | **Unvaccinated (n=164)** |
| --- | --- | --- |
| <15 | 80 (25.0%) | 43 (26.2%) |
| 15 to 20 | 139 (43.4%) | 63 (38.4%) |
| 20 to 25 | 82 (25.6%) | 37 (22.6%) |
| 25 to 30 | 19 (5.9%) | 21 (12.8%) |

**Supplemental Table 5.** Accession numbers added to the Global Influenza Surveillance & Response System (GISAID) EpiCoV database

| **Accession numbers** |  |  |  |  |  |  |  |  |
| --- | --- | --- | --- | --- | --- | --- | --- | --- |
| EPI_ISL_2507342 | EPI_ISL_2942057 | EPI_ISL_3273415 | EPI_ISL_5522964 | EPI_ISL_5523130 | EPI_ISL_5523490 | EPI_ISL_5523682 | EPI_ISL_5523818 | EPI_ISL_5572677 |
| EPI_ISL_2507343 | EPI_ISL_2942058 | EPI_ISL_3273416 | EPI_ISL_5522965 | EPI_ISL_5523131 | EPI_ISL_5523491 | EPI_ISL_5523683 | EPI_ISL_5523819 | EPI_ISL_5572693 |
| EPI_ISL_2507344 | EPI_ISL_2942059 | EPI_ISL_3273417 | EPI_ISL_5522966 | EPI_ISL_5523133 | EPI_ISL_5523492 | EPI_ISL_5523684 | EPI_ISL_5523820 | EPI_ISL_5572704 |
| EPI_ISL_2507345 | EPI_ISL_2942060 | EPI_ISL_3273418 | EPI_ISL_5522967 | EPI_ISL_5523134 | EPI_ISL_5523493 | EPI_ISL_5523685 | EPI_ISL_5523821 | EPI_ISL_5572705 |
| EPI_ISL_2507346 | EPI_ISL_2942061 | EPI_ISL_3273419 | EPI_ISL_5522968 | EPI_ISL_5523135 | EPI_ISL_5523494 | EPI_ISL_5523686 | EPI_ISL_5523822 | EPI_ISL_5572708 |
| EPI_ISL_2755404 | EPI_ISL_2942062 | EPI_ISL_3273420 | EPI_ISL_5522969 | EPI_ISL_5523136 | EPI_ISL_5523495 | EPI_ISL_5523687 | EPI_ISL_5523823 | EPI_ISL_5572717 |
| EPI_ISL_2755405 | EPI_ISL_2942063 | EPI_ISL_3273421 | EPI_ISL_5522970 | EPI_ISL_5523137 | EPI_ISL_5523496 | EPI_ISL_5523688 | EPI_ISL_5523824 | EPI_ISL_5572731 |
| EPI_ISL_2755406 | EPI_ISL_2942064 | EPI_ISL_3273422 | EPI_ISL_5522971 | EPI_ISL_5523138 | EPI_ISL_5523497 | EPI_ISL_5523689 | EPI_ISL_5523825 | EPI_ISL_5572734 |
| EPI_ISL_2755407 | EPI_ISL_2942065 | EPI_ISL_3273423 | EPI_ISL_5522972 | EPI_ISL_5523139 | EPI_ISL_5523498 | EPI_ISL_5523690 | EPI_ISL_5523826 | EPI_ISL_5572740 |
| EPI_ISL_2755408 | EPI_ISL_2942066 | EPI_ISL_3273424 | EPI_ISL_5522973 | EPI_ISL_5523140 | EPI_ISL_5523499 | EPI_ISL_5523691 | EPI_ISL_5523827 | EPI_ISL_5572751 |
| EPI_ISL_2755409 | EPI_ISL_2942067 | EPI_ISL_3273425 | EPI_ISL_5522974 | EPI_ISL_5523141 | EPI_ISL_5523500 | EPI_ISL_5523692 | EPI_ISL_5523828 | EPI_ISL_5572758 |
| EPI_ISL_2755410 | EPI_ISL_2942068 | EPI_ISL_3273426 | EPI_ISL_5522975 | EPI_ISL_5523142 | EPI_ISL_5523501 | EPI_ISL_5523693 | EPI_ISL_5523829 | EPI_ISL_5572760 |
| EPI_ISL_2755411 | EPI_ISL_2942069 | EPI_ISL_3273427 | EPI_ISL_5522976 | EPI_ISL_5523143 | EPI_ISL_5523502 | EPI_ISL_5523694 | EPI_ISL_5523830 | EPI_ISL_5572779 |
| EPI_ISL_2783654 | EPI_ISL_2942070 | EPI_ISL_3273428 | EPI_ISL_5522977 | EPI_ISL_5523144 | EPI_ISL_5523503 | EPI_ISL_5523695 | EPI_ISL_5523831 | EPI_ISL_5572788 |
| EPI_ISL_2783655 | EPI_ISL_2942071 | EPI_ISL_5522816 | EPI_ISL_5522979 | EPI_ISL_5523145 | EPI_ISL_5523504 | EPI_ISL_5523696 | EPI_ISL_5523832 | EPI_ISL_5572792 |
| EPI_ISL_2783656 | EPI_ISL_2942072 | EPI_ISL_5522817 | EPI_ISL_5522980 | EPI_ISL_5523146 | EPI_ISL_5523505 | EPI_ISL_5523697 | EPI_ISL_5523833 | EPI_ISL_5572800 |
| EPI_ISL_2783657 | EPI_ISL_2942073 | EPI_ISL_5522818 | EPI_ISL_5522981 | EPI_ISL_5523147 | EPI_ISL_5523506 | EPI_ISL_5523698 | EPI_ISL_5523834 | EPI_ISL_5572802 |
| EPI_ISL_2783658 | EPI_ISL_2942074 | EPI_ISL_5522819 | EPI_ISL_5522983 | EPI_ISL_5523149 | EPI_ISL_5523507 | EPI_ISL_5523699 | EPI_ISL_5523835 | EPI_ISL_5572811 |
| EPI_ISL_2783659 | EPI_ISL_2942075 | EPI_ISL_5522820 | EPI_ISL_5522984 | EPI_ISL_5523150 | EPI_ISL_5523508 | EPI_ISL_5523700 | EPI_ISL_5523836 | EPI_ISL_5572820 |
| EPI_ISL_2783660 | EPI_ISL_2942076 | EPI_ISL_5522821 | EPI_ISL_5522985 | EPI_ISL_5523151 | EPI_ISL_5523509 | EPI_ISL_5523701 | EPI_ISL_5523837 | EPI_ISL_5572828 |
| EPI_ISL_2783661 | EPI_ISL_2942077 | EPI_ISL_5522822 | EPI_ISL_5522986 | EPI_ISL_5523152 | EPI_ISL_5523510 | EPI_ISL_5523702 | EPI_ISL_5523838 | EPI_ISL_5572830 |
| EPI_ISL_2783662 | EPI_ISL_2942078 | EPI_ISL_5522823 | EPI_ISL_5522987 | EPI_ISL_5523153 | EPI_ISL_5523511 | EPI_ISL_5523703 | EPI_ISL_5523839 | EPI_ISL_5572837 |
| EPI_ISL_2783663 | EPI_ISL_2942079 | EPI_ISL_5522824 | EPI_ISL_5522988 | EPI_ISL_5523154 | EPI_ISL_5523512 | EPI_ISL_5523704 | EPI_ISL_5523840 | EPI_ISL_5572851 |
| EPI_ISL_2783664 | EPI_ISL_2942080 | EPI_ISL_5522825 | EPI_ISL_5522989 | EPI_ISL_5523155 | EPI_ISL_5523513 | EPI_ISL_5523705 | EPI_ISL_5523841 | EPI_ISL_5572858 |
| EPI_ISL_2783975 | EPI_ISL_2942081 | EPI_ISL_5522826 | EPI_ISL_5522990 | EPI_ISL_5523156 | EPI_ISL_5523514 | EPI_ISL_5523706 | EPI_ISL_5523898 | EPI_ISL_5572860 |
| EPI_ISL_2783976 | EPI_ISL_2942082 | EPI_ISL_5522827 | EPI_ISL_5522991 | EPI_ISL_5523157 | EPI_ISL_5523515 | EPI_ISL_5523707 | EPI_ISL_5523899 | EPI_ISL_5572865 |
| EPI_ISL_2783978 | EPI_ISL_2942083 | EPI_ISL_5522828 | EPI_ISL_5522992 | EPI_ISL_5523158 | EPI_ISL_5523516 | EPI_ISL_5523708 | EPI_ISL_5523900 | EPI_ISL_5572874 |
| EPI_ISL_2783979 | EPI_ISL_2942084 | EPI_ISL_5522829 | EPI_ISL_5522993 | EPI_ISL_5523159 | EPI_ISL_5523517 | EPI_ISL_5523737 | EPI_ISL_5523901 | EPI_ISL_5572886 |
| EPI_ISL_2783980 | EPI_ISL_2942085 | EPI_ISL_5522830 | EPI_ISL_5522994 | EPI_ISL_5523160 | EPI_ISL_5523518 | EPI_ISL_5523739 | EPI_ISL_5523902 | EPI_ISL_5572905 |
| EPI_ISL_2783981 | EPI_ISL_2942086 | EPI_ISL_5522831 | EPI_ISL_5522995 | EPI_ISL_5523161 | EPI_ISL_5523519 | EPI_ISL_5523740 | EPI_ISL_5523903 | EPI_ISL_5572923 |
| EPI_ISL_2783982 | EPI_ISL_2942087 | EPI_ISL_5522832 | EPI_ISL_5522996 | EPI_ISL_5523163 | EPI_ISL_5523520 | EPI_ISL_5523741 | EPI_ISL_5523904 | EPI_ISL_5572928 |
| EPI_ISL_2783983 | EPI_ISL_2942088 | EPI_ISL_5522833 | EPI_ISL_5522997 | EPI_ISL_5523164 | EPI_ISL_5523521 | EPI_ISL_5523742 | EPI_ISL_5523905 | EPI_ISL_5572934 |
| EPI_ISL_2783985 | EPI_ISL_2942089 | EPI_ISL_5522834 | EPI_ISL_5522998 | EPI_ISL_5523165 | EPI_ISL_5523522 | EPI_ISL_5523743 | EPI_ISL_5523906 | EPI_ISL_5572936 |
| EPI_ISL_2784318 | EPI_ISL_2942090 | EPI_ISL_5522835 | EPI_ISL_5523000 | EPI_ISL_5523166 | EPI_ISL_5523523 | EPI_ISL_5523744 | EPI_ISL_5523907 | EPI_ISL_5572956 |
| EPI_ISL_2784319 | EPI_ISL_2942091 | EPI_ISL_5522836 | EPI_ISL_5523001 | EPI_ISL_5523167 | EPI_ISL_5523524 | EPI_ISL_5523745 | EPI_ISL_5523908 | EPI_ISL_5572979 |
| EPI_ISL_2784320 | EPI_ISL_2942092 | EPI_ISL_5522837 | EPI_ISL_5523002 | EPI_ISL_5523168 | EPI_ISL_5523525 | EPI_ISL_5523746 | EPI_ISL_5523909 | EPI_ISL_5572983 |
| EPI_ISL_2784321 | EPI_ISL_2942093 | EPI_ISL_5522838 | EPI_ISL_5523003 | EPI_ISL_5523169 | EPI_ISL_5523526 | EPI_ISL_5523747 | EPI_ISL_5523910 | EPI_ISL_5572988 |
| EPI_ISL_2784322 | EPI_ISL_2942094 | EPI_ISL_5522839 | EPI_ISL_5523060 | EPI_ISL_5523170 | EPI_ISL_5523527 | EPI_ISL_5523748 | EPI_ISL_5523911 | EPI_ISL_5573002 |
| EPI_ISL_2784323 | EPI_ISL_2942095 | EPI_ISL_5522840 | EPI_ISL_5523061 | EPI_ISL_5523171 | EPI_ISL_5523528 | EPI_ISL_5523749 | EPI_ISL_5523912 | EPI_ISL_5573037 |
| EPI_ISL_2784324 | EPI_ISL_2942096 | EPI_ISL_5522841 | EPI_ISL_5523063 | EPI_ISL_5523172 | EPI_ISL_5523529 | EPI_ISL_5523750 | EPI_ISL_5523913 | EPI_ISL_5573051 |
| EPI_ISL_2784325 | EPI_ISL_3064548 | EPI_ISL_5522842 | EPI_ISL_5523064 | EPI_ISL_5523173 | EPI_ISL_5523530 | EPI_ISL_5523751 | EPI_ISL_5523914 | EPI_ISL_5573053 |
| EPI_ISL_2784326 | EPI_ISL_3064549 | EPI_ISL_5522843 | EPI_ISL_5523065 | EPI_ISL_5523174 | EPI_ISL_5523531 | EPI_ISL_5523752 | EPI_ISL_5523915 | EPI_ISL_5573063 |
| EPI_ISL_2784327 | EPI_ISL_3064550 | EPI_ISL_5522844 | EPI_ISL_5523066 | EPI_ISL_5523175 | EPI_ISL_5523532 | EPI_ISL_5523753 | EPI_ISL_5523916 | EPI_ISL_5573082 |
| EPI_ISL_2839961 | EPI_ISL_3064551 | EPI_ISL_5522845 | EPI_ISL_5523067 | EPI_ISL_5523176 | EPI_ISL_5523533 | EPI_ISL_5523754 | EPI_ISL_5523917 | EPI_ISL_5573105 |
| EPI_ISL_2839962 | EPI_ISL_3064552 | EPI_ISL_5522846 | EPI_ISL_5523068 | EPI_ISL_5523177 | EPI_ISL_5523622 | EPI_ISL_5523755 | EPI_ISL_5523918 | EPI_ISL_5573107 |
| EPI_ISL_2839963 | EPI_ISL_3064553 | EPI_ISL_5522847 | EPI_ISL_5523069 | EPI_ISL_5523178 | EPI_ISL_5523623 | EPI_ISL_5523756 | EPI_ISL_5523919 | EPI_ISL_5573110 |
| EPI_ISL_2839964 | EPI_ISL_3064554 | EPI_ISL_5522848 | EPI_ISL_5523070 | EPI_ISL_5523179 | EPI_ISL_5523624 | EPI_ISL_5523757 | EPI_ISL_5523920 | EPI_ISL_5573137 |
| EPI_ISL_2839965 | EPI_ISL_3064555 | EPI_ISL_5522849 | EPI_ISL_5523071 | EPI_ISL_5523181 | EPI_ISL_5523625 | EPI_ISL_5523758 | EPI_ISL_5523921 | EPI_ISL_5573149 |
| EPI_ISL_2839966 | EPI_ISL_3064556 | EPI_ISL_5522850 | EPI_ISL_5523072 | EPI_ISL_5523182 | EPI_ISL_5523626 | EPI_ISL_5523759 | EPI_ISL_5523922 | EPI_ISL_5573209 |
| EPI_ISL_2839967 | EPI_ISL_3064557 | EPI_ISL_5522851 | EPI_ISL_5523073 | EPI_ISL_5523183 | EPI_ISL_5523627 | EPI_ISL_5523760 | EPI_ISL_5523923 | EPI_ISL_5573216 |
| EPI_ISL_2839968 | EPI_ISL_3064558 | EPI_ISL_5522852 | EPI_ISL_5523074 | EPI_ISL_5523184 | EPI_ISL_5523628 | EPI_ISL_5523761 | EPI_ISL_5523924 | EPI_ISL_5573218 |
| EPI_ISL_2839969 | EPI_ISL_3064559 | EPI_ISL_5522853 | EPI_ISL_5523075 | EPI_ISL_5523185 | EPI_ISL_5523629 | EPI_ISL_5523762 | EPI_ISL_5523925 | EPI_ISL_5573220 |
| EPI_ISL_2839970 | EPI_ISL_3064560 | EPI_ISL_5522854 | EPI_ISL_5523076 | EPI_ISL_5523186 | EPI_ISL_5523630 | EPI_ISL_5523763 | EPI_ISL_5523926 | EPI_ISL_5573244 |
| EPI_ISL_2839971 | EPI_ISL_3064561 | EPI_ISL_5522855 | EPI_ISL_5523077 | EPI_ISL_5523187 | EPI_ISL_5523631 | EPI_ISL_5523770 | EPI_ISL_5523927 | EPI_ISL_5573247 |
| EPI_ISL_2839972 | EPI_ISL_3064562 | EPI_ISL_5522856 | EPI_ISL_5523078 | EPI_ISL_5523188 | EPI_ISL_5523632 | EPI_ISL_5523771 | EPI_ISL_5523928 | EPI_ISL_5573260 |
| EPI_ISL_2839973 | EPI_ISL_3064563 | EPI_ISL_5522857 | EPI_ISL_5523079 | EPI_ISL_5523189 | EPI_ISL_5523633 | EPI_ISL_5523772 | EPI_ISL_5523929 | EPI_ISL_5573265 |
| EPI_ISL_2839974 | EPI_ISL_3064564 | EPI_ISL_5522858 | EPI_ISL_5523080 | EPI_ISL_5523190 | EPI_ISL_5523634 | EPI_ISL_5523773 | EPI_ISL_5523930 | EPI_ISL_5573271 |
| EPI_ISL_2839975 | EPI_ISL_3064565 | EPI_ISL_5522859 | EPI_ISL_5523081 | EPI_ISL_5523191 | EPI_ISL_5523635 | EPI_ISL_5523774 | EPI_ISL_5523931 | EPI_ISL_5573278 |
| EPI_ISL_2839976 | EPI_ISL_3064566 | EPI_ISL_5522860 | EPI_ISL_5523082 | EPI_ISL_5523192 | EPI_ISL_5523636 | EPI_ISL_5523775 | EPI_ISL_5523932 | EPI_ISL_5573280 |
| EPI_ISL_2839977 | EPI_ISL_3064567 | EPI_ISL_5522861 | EPI_ISL_5523083 | EPI_ISL_5523193 | EPI_ISL_5523637 | EPI_ISL_5523776 | EPI_ISL_5523933 | EPI_ISL_5573287 |
| EPI_ISL_2839978 | EPI_ISL_3064568 | EPI_ISL_5522862 | EPI_ISL_5523084 | EPI_ISL_5523194 | EPI_ISL_5523638 | EPI_ISL_5523777 | EPI_ISL_5523934 | EPI_ISL_5573289 |
| EPI_ISL_2942016 | EPI_ISL_3064569 | EPI_ISL_5522923 | EPI_ISL_5523085 | EPI_ISL_5523195 | EPI_ISL_5523639 | EPI_ISL_5523778 | EPI_ISL_5523935 | EPI_ISL_5573302 |
| EPI_ISL_2942017 | EPI_ISL_3064570 | EPI_ISL_5522924 | EPI_ISL_5523088 | EPI_ISL_5523451 | EPI_ISL_5523640 | EPI_ISL_5523779 | EPI_ISL_5523936 | EPI_ISL_5573307 |
| EPI_ISL_2942018 | EPI_ISL_3064571 | EPI_ISL_5522925 | EPI_ISL_5523089 | EPI_ISL_5523452 | EPI_ISL_5523641 | EPI_ISL_5523780 | EPI_ISL_5523937 | EPI_ISL_5573317 |
| EPI_ISL_2942019 | EPI_ISL_3064572 | EPI_ISL_5522926 | EPI_ISL_5523091 | EPI_ISL_5523453 | EPI_ISL_5523642 | EPI_ISL_5523781 | EPI_ISL_5523938 | EPI_ISL_5573328 |
| EPI_ISL_2942021 | EPI_ISL_3064573 | EPI_ISL_5522927 | EPI_ISL_5523092 | EPI_ISL_5523454 | EPI_ISL_5523643 | EPI_ISL_5523782 | EPI_ISL_5523939 | EPI_ISL_5573337 |
| EPI_ISL_2942022 | EPI_ISL_3064574 | EPI_ISL_5522928 | EPI_ISL_5523093 | EPI_ISL_5523455 | EPI_ISL_5523644 | EPI_ISL_5523783 | EPI_ISL_5523940 | EPI_ISL_5573342 |
| EPI_ISL_2942023 | EPI_ISL_3064575 | EPI_ISL_5522929 | EPI_ISL_5523094 | EPI_ISL_5523456 | EPI_ISL_5523645 | EPI_ISL_5523784 | EPI_ISL_5523941 | EPI_ISL_5573353 |
| EPI_ISL_2942024 | EPI_ISL_3064576 | EPI_ISL_5522930 | EPI_ISL_5523095 | EPI_ISL_5523457 | EPI_ISL_5523646 | EPI_ISL_5523785 | EPI_ISL_5523942 | EPI_ISL_5573359 |
| EPI_ISL_2942025 | EPI_ISL_3064577 | EPI_ISL_5522931 | EPI_ISL_5523096 | EPI_ISL_5523458 | EPI_ISL_5523647 | EPI_ISL_5523786 | EPI_ISL_5523943 | EPI_ISL_5573364 |
| EPI_ISL_2942026 | EPI_ISL_3064578 | EPI_ISL_5522932 | EPI_ISL_5523097 | EPI_ISL_5523459 | EPI_ISL_5523648 | EPI_ISL_5523787 | EPI_ISL_5523944 | EPI_ISL_5573376 |
| EPI_ISL_2942027 | EPI_ISL_3064579 | EPI_ISL_5522933 | EPI_ISL_5523098 | EPI_ISL_5523460 | EPI_ISL_5523652 | EPI_ISL_5523788 | EPI_ISL_5523945 | EPI_ISL_5573391 |
| EPI_ISL_2942028 | EPI_ISL_3064580 | EPI_ISL_5522934 | EPI_ISL_5523099 | EPI_ISL_5523461 | EPI_ISL_5523653 | EPI_ISL_5523789 | EPI_ISL_5523946 | EPI_ISL_5573398 |
| EPI_ISL_2942029 | EPI_ISL_3064581 | EPI_ISL_5522935 | EPI_ISL_5523100 | EPI_ISL_5523462 | EPI_ISL_5523654 | EPI_ISL_5523790 | EPI_ISL_5523947 | EPI_ISL_5573404 |
| EPI_ISL_2942030 | EPI_ISL_3064582 | EPI_ISL_5522936 | EPI_ISL_5523101 | EPI_ISL_5523463 | EPI_ISL_5523655 | EPI_ISL_5523791 | EPI_ISL_5523948 | EPI_ISL_5573406 |
| EPI_ISL_2942031 | EPI_ISL_3064583 | EPI_ISL_5522937 | EPI_ISL_5523102 | EPI_ISL_5523464 | EPI_ISL_5523656 | EPI_ISL_5523792 | EPI_ISL_5523949 | EPI_ISL_5573408 |
| EPI_ISL_2942032 | EPI_ISL_3064584 | EPI_ISL_5522938 | EPI_ISL_5523103 | EPI_ISL_5523465 | EPI_ISL_5523657 | EPI_ISL_5523793 | EPI_ISL_5523950 | EPI_ISL_5573418 |
| EPI_ISL_2942033 | EPI_ISL_3064585 | EPI_ISL_5522939 | EPI_ISL_5523104 | EPI_ISL_5523466 | EPI_ISL_5523658 | EPI_ISL_5523794 | EPI_ISL_5523951 |  |
| EPI_ISL_2942034 | EPI_ISL_3064586 | EPI_ISL_5522940 | EPI_ISL_5523105 | EPI_ISL_5523467 | EPI_ISL_5523659 | EPI_ISL_5523795 | EPI_ISL_5523952 |  |
| EPI_ISL_2942035 | EPI_ISL_3064587 | EPI_ISL_5522941 | EPI_ISL_5523107 | EPI_ISL_5523468 | EPI_ISL_5523660 | EPI_ISL_5523796 | EPI_ISL_5523953 |  |
| EPI_ISL_2942036 | EPI_ISL_3064588 | EPI_ISL_5522942 | EPI_ISL_5523108 | EPI_ISL_5523469 | EPI_ISL_5523661 | EPI_ISL_5523797 | EPI_ISL_5523954 |  |
| EPI_ISL_2942037 | EPI_ISL_3064589 | EPI_ISL_5522943 | EPI_ISL_5523109 | EPI_ISL_5523470 | EPI_ISL_5523662 | EPI_ISL_5523798 | EPI_ISL_5523955 |  |
| EPI_ISL_2942038 | EPI_ISL_3064590 | EPI_ISL_5522944 | EPI_ISL_5523110 | EPI_ISL_5523471 | EPI_ISL_5523663 | EPI_ISL_5523799 | EPI_ISL_5523956 |  |
| EPI_ISL_2942039 | EPI_ISL_3064591 | EPI_ISL_5522945 | EPI_ISL_5523111 | EPI_ISL_5523472 | EPI_ISL_5523664 | EPI_ISL_5523800 | EPI_ISL_5523957 |  |
| EPI_ISL_2942040 | EPI_ISL_3064592 | EPI_ISL_5522946 | EPI_ISL_5523112 | EPI_ISL_5523473 | EPI_ISL_5523665 | EPI_ISL_5523801 | EPI_ISL_5523958 |  |
| EPI_ISL_2942041 | EPI_ISL_3064593 | EPI_ISL_5522947 | EPI_ISL_5523113 | EPI_ISL_5523474 | EPI_ISL_5523666 | EPI_ISL_5523802 | EPI_ISL_5523959 |  |
| EPI_ISL_2942042 | EPI_ISL_3064594 | EPI_ISL_5522948 | EPI_ISL_5523114 | EPI_ISL_5523475 | EPI_ISL_5523667 | EPI_ISL_5523803 | EPI_ISL_5523960 |  |
| EPI_ISL_2942043 | EPI_ISL_3064595 | EPI_ISL_5522949 | EPI_ISL_5523115 | EPI_ISL_5523476 | EPI_ISL_5523668 | EPI_ISL_5523804 | EPI_ISL_5523961 |  |
| EPI_ISL_2942044 | EPI_ISL_3064596 | EPI_ISL_5522950 | EPI_ISL_5523116 | EPI_ISL_5523477 | EPI_ISL_5523669 | EPI_ISL_5523805 | EPI_ISL_5523962 |  |
| EPI_ISL_2942045 | EPI_ISL_3064597 | EPI_ISL_5522951 | EPI_ISL_5523118 | EPI_ISL_5523478 | EPI_ISL_5523670 | EPI_ISL_5523806 | EPI_ISL_5523963 |  |
| EPI_ISL_2942046 | EPI_ISL_3270120 | EPI_ISL_5522952 | EPI_ISL_5523119 | EPI_ISL_5523479 | EPI_ISL_5523671 | EPI_ISL_5523807 | EPI_ISL_5523964 |  |
| EPI_ISL_2942047 | EPI_ISL_3270183 | EPI_ISL_5522953 | EPI_ISL_5523120 | EPI_ISL_5523480 | EPI_ISL_5523672 | EPI_ISL_5523808 | EPI_ISL_5523965 |  |
| EPI_ISL_2942048 | EPI_ISL_3270199 | EPI_ISL_5522954 | EPI_ISL_5523121 | EPI_ISL_5523481 | EPI_ISL_5523673 | EPI_ISL_5523809 | EPI_ISL_5523966 |  |
| EPI_ISL_2942049 | EPI_ISL_3270426 | EPI_ISL_5522955 | EPI_ISL_5523122 | EPI_ISL_5523482 | EPI_ISL_5523674 | EPI_ISL_5523810 | EPI_ISL_5523970 |  |
| EPI_ISL_2942050 | EPI_ISL_3270427 | EPI_ISL_5522956 | EPI_ISL_5523123 | EPI_ISL_5523483 | EPI_ISL_5523675 | EPI_ISL_5523811 | EPI_ISL_5523971 |  |
| EPI_ISL_2942051 | EPI_ISL_3273409 | EPI_ISL_5522957 | EPI_ISL_5523124 | EPI_ISL_5523484 | EPI_ISL_5523676 | EPI_ISL_5523812 | EPI_ISL_5523972 |  |
| EPI_ISL_2942052 | EPI_ISL_3273410 | EPI_ISL_5522958 | EPI_ISL_5523125 | EPI_ISL_5523485 | EPI_ISL_5523677 | EPI_ISL_5523813 | EPI_ISL_5523973 |  |
| EPI_ISL_2942053 | EPI_ISL_3273411 | EPI_ISL_5522959 | EPI_ISL_5523126 | EPI_ISL_5523486 | EPI_ISL_5523678 | EPI_ISL_5523814 | EPI_ISL_5523974 |  |
| EPI_ISL_2942054 | EPI_ISL_3273412 | EPI_ISL_5522960 | EPI_ISL_5523127 | EPI_ISL_5523487 | EPI_ISL_5523679 | EPI_ISL_5523815 | EPI_ISL_5523975 |  |
| EPI_ISL_2942055 | EPI_ISL_3273413 | EPI_ISL_5522961 | EPI_ISL_5523128 | EPI_ISL_5523488 | EPI_ISL_5523680 | EPI_ISL_5523816 | EPI_ISL_5572659 |  |
| EPI_ISL_2942056 | EPI_ISL_3273414 | EPI_ISL_5522962 | EPI_ISL_5523129 | EPI_ISL_5523489 | EPI_ISL_5523681 | EPI_ISL_5523817 | EPI_ISL_5572671 |  |

**Supplemental Table Figure 1a**. SARS-CoV-2 lineage data by variant over time from a cohort of outpatient vaccinated participants in Los Angeles County and Riverside County, California, May 27, 2021 to September 9, 2021 (n= 348)

**Supplemental Table Figure 1b.** SARS-CoV-2 lineage data by variant over time from a cohort of outpatient unvaccinated participants in Los Angeles County and Riverside County, California, May 27, 2021 to September 9, 2021 (n= 177)


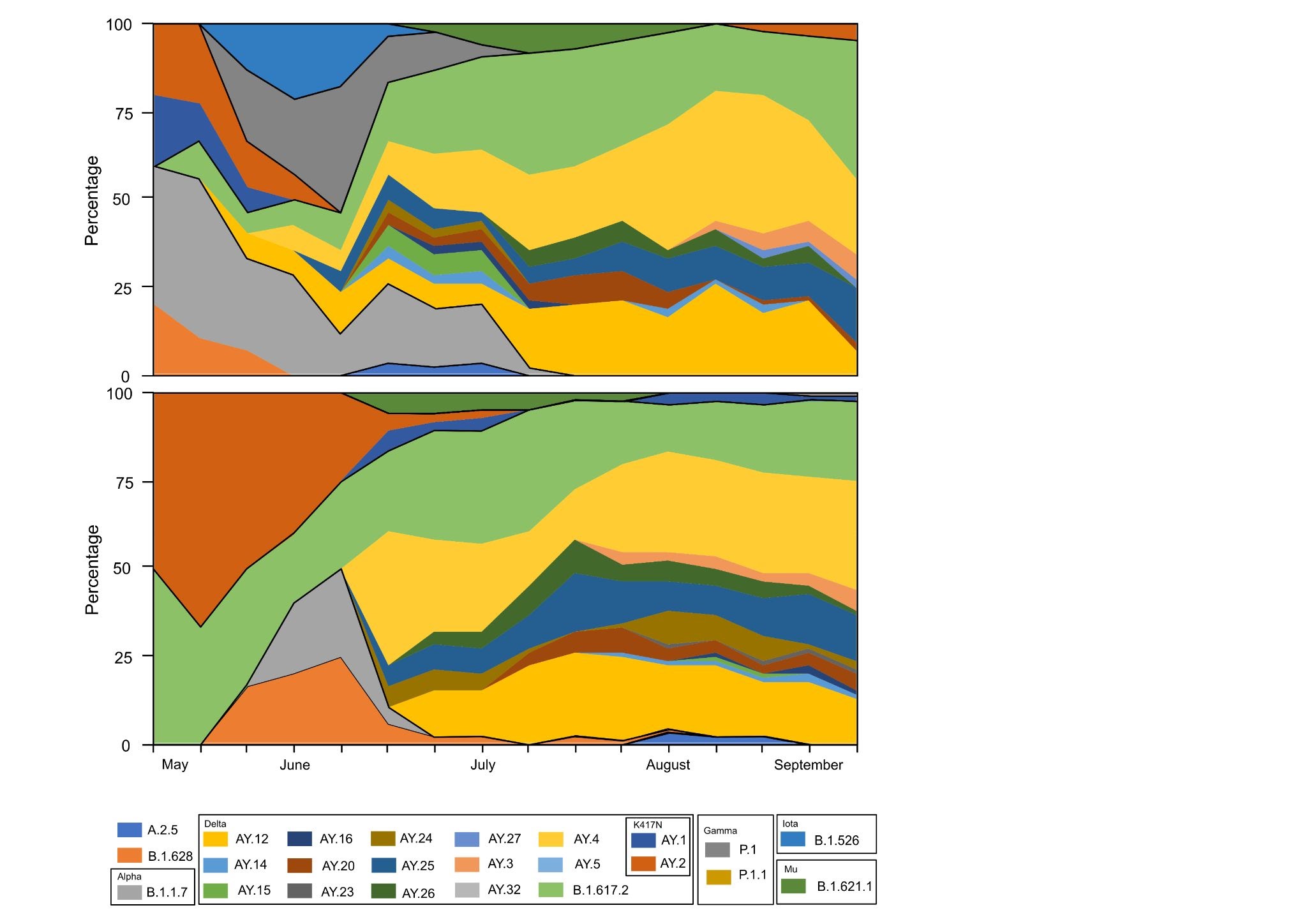


**Supplemental Figure 2.** Hallmark Spike Protein mutations in variants of concern and mutations found in the isolate hCoV-19/USA/CA-Curative-707962712299/2021
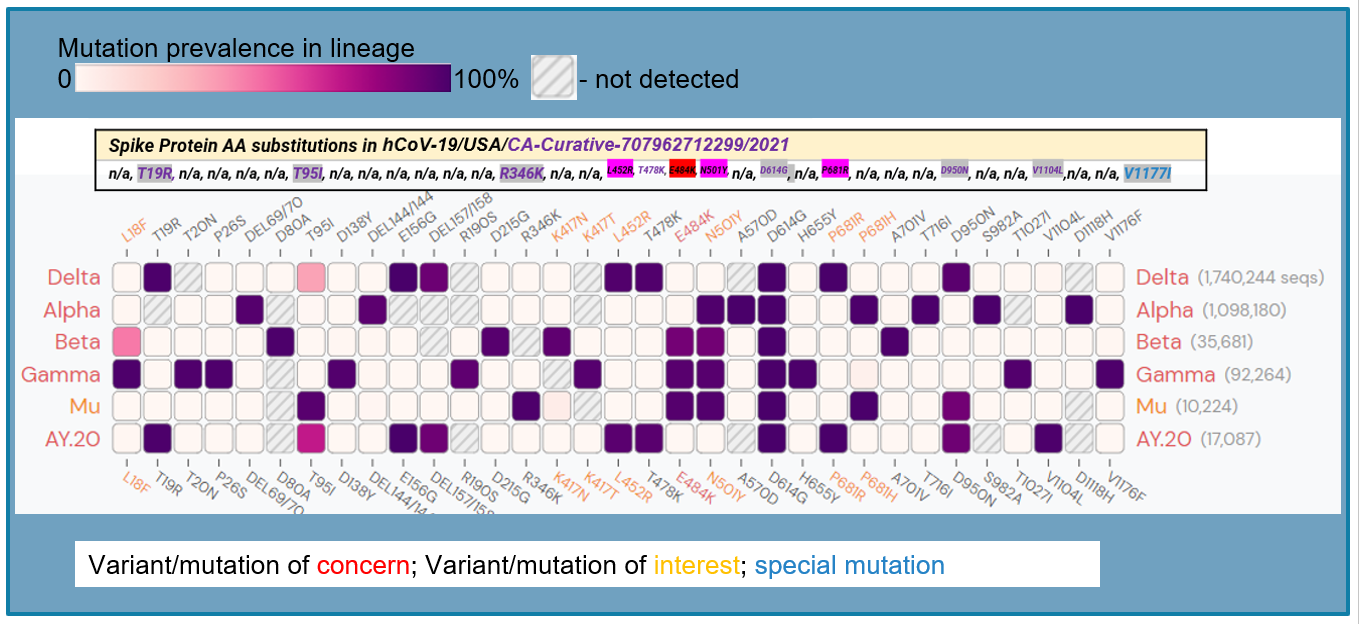


*Lineage comparison: Alaa Abdel Latif, Julia L. Mullen, Manar Alkuzweny, Ginger Tsueng, Marco Cano, Emily Haag, Jerry Zhou, Mark Zeller, Emory Hufbauer, Nate Matteson, Chunlei Wu, Kristian G. Andersen, Andrew I. Su, Karthik Gangavarapu, Laura D. Hughes, and the Center for Viral Systems Biology. outbreak.info, (available at https://outbreak.info/compare-lineages?gene=N&gene=S&threshold=75&dark=true). Accessed 20 October 2021.

**Supplemental Figure 3a.** SARS-CoV-2 lineages with over 5% prevalence for the Spike Protein V1104L mutation.

**Supplemental Figure 3b.** The AY.20 SARS-CoV-2 lineage uniquely carries both mutations: S:V1104L with at least 5% prevalence and S:V1177I mutation with at least 1% prevalence
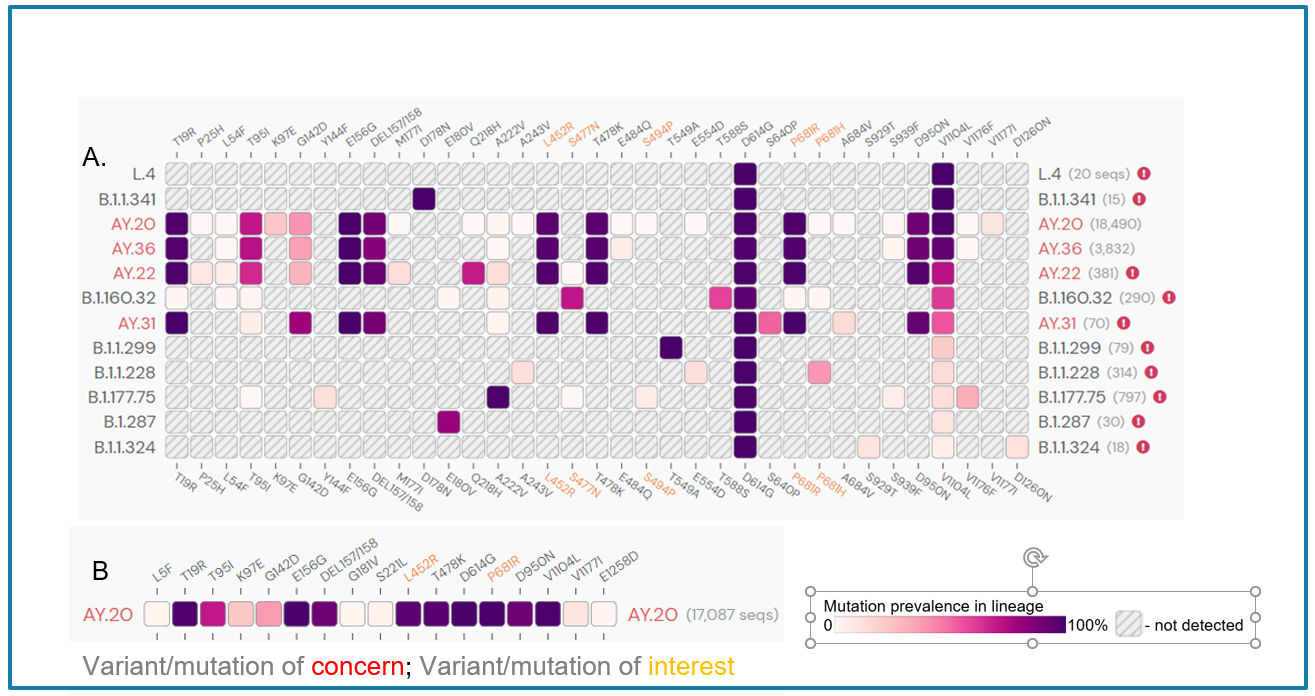


Supplemental Figure 3a: *Lineage Comparison. Alaa Abdel Latif, Julia L. Mullen, Manar Alkuzweny, Ginger Tsueng, Marco Cano, Emily Haag, Jerry Zhou, Mark Zeller, Emory Hufbauer, Nate Matteson, Chunlei Wu, Kristian G. Andersen, Andrew I. Su, Karthik Gangavarapu, Laura D. Hughes, and the Center for Viral Systems Biology. outbreak.info, (available at https://outbreak.info/compare-lineages?pango=AY.20&pango=AY.25&gene=S&threshold=75&dark=false). Accessed 15 October 2021. **The only lineage that carries both S:V1104L and S:V1177I mutations is AY.20. S:V1104L and S:V1177I prevalence in AY.20 is 98.4% and 9.4% respectively.

Supplemental Figure 3b: *the search was done for any of two mutation prevalence at least 5% and strain prevalence selected at least at 0.001% over the last 60-600 days

**Supplemental Figure 4.** SARS-CoV-2 lineages that show over 5% prevalence for V1104L mutation in the Spike Protein **
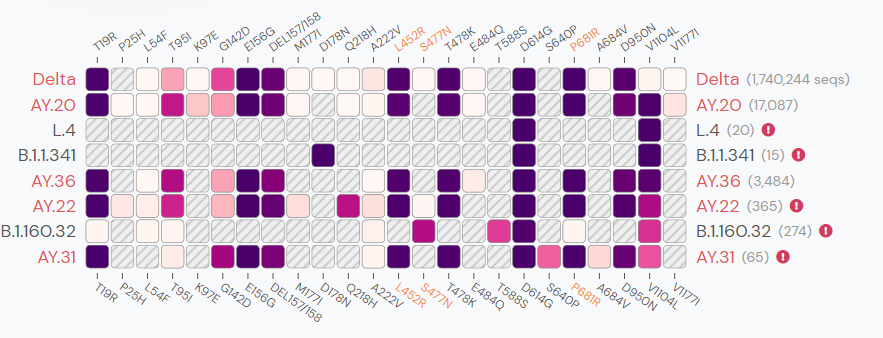
**

*The only lineage that carries both S:V1104L and S:V1177I mutations is AY20 (delta). S:V1104L and S:V1177I prevalence in AY.20 is 99.1% and 9.5% respectively.
